# 3D Printed Functionalized Nanocellulose as an Adsorbent in Batch and Fixed-Bed Systems

**DOI:** 10.3390/polym15040969

**Published:** 2023-02-15

**Authors:** Mohd Shaiful Sajab, Wan Nazihah Liyana Wan Jusoh, Denesh Mohan, Hatika Kaco, Rubiyah Baini

**Affiliations:** 1 Research Center for Sustainable Process Technology (CESPRO), Faculty of Engineering and Built Environment, Universiti Kebangsaan Malaysia, Bangi 43600, Selangor, Malaysia; 2 Department of Chemical and Process Engineering, Faculty of Engineering and Built Environment, Universiti Kebangsaan Malaysia, Bangi 43600, Selangor, Malaysia; 3 Kolej GENIUS Insan, Universiti Sains Islam Malaysia, Bandar Baru Nilai, Nilai 71800, Negeri Sembilan, Malaysia; 4 Faculty of Engineering, Universiti Malaysia Sarawak (UNIMAS), Kota Samarahan 94300, Sarawak, Malaysia

**Keywords:** 3D printing, additive manufacturing, cellulose, water remediation

## Abstract

Nanocellulose, a refined form of cellulose, can be further functionalized on surface-active sites, with a catalyst as a regenerative agent. Newly developed adsorbents are expected to have the characteristics of good and rapid adsorption performance and regeneration properties with flexible structure using 3D printing technology. In this work, the adsorption performance of 3D printed functionalized nanocellulose was investigated using batch and fixed-bed column adsorption. Kinetics adsorption studies were divided into different adsorption models, with the pseudo-second order model showing a better correlation coefficient than the pseudo-first order and intraparticle diffusion models. The Langmuir and Thomas models were used to calculate the adsorption performance of batch and fixed-bed columns. Given the catalytic activity of Fenton oxidation, the fixed-bed column was regenerated up to five adsorption-desorption cycles, suggesting satisfactory performance of the column, with a slightly reduced adsorption capacity.

## 1. Introduction

Every year, a large quantity of synthetic dyes, approximately 8 × 10^5^ tons, are produced. However, they exhibit significant losses, with 15% of the production discharged during application [1]. Dyes can have harmful effects on humans, including causing dermatitis, affecting the central nervous system, and leading to long-term genotoxicity and cancer. Additionally, dyes and pigments can indirectly impact human health by flowing through the food chain, ultimately being consumed by humans. Research has shown that their toxicity is magnified by a factor of 1000 when detected in humans compared to the actual concentration in the food chain [2,3].

To mitigate these impacts, proper treatment for decolorization is necessary, as traditional sewage treatment methods are inadequate for removing dyes to the extent required for meeting minimum color standards. Currently, there are several techniques for treating wastewater, which can be broadly divided into two categories: physicochemical and biological treatments. Treating dyes can be expensive and can involve long and complicated processes, which can also generate secondary hazardous materials [3,4]. Each treatment method has its own advantages and disadvantages, and its limitations depend on the specific conditions of the effluent. A more effective, sustainable, and environmentally friendly alternative method is desired for the treatment process.

To address this challenge, low-cost alternative adsorbents have been developed, such as those made from clays, siliceous minerals, agricultural waste, biosorbents, and other materials. These have been shown to be as effective as commercially available adsorbents. Lignocellulosic biomass derived from agricultural waste is the most abundant and readily accessible raw material. Modifying it into a nano-sized form, such as nanocellulose, leads to a rapid uptake of the adsorbate [5,6].

Owing to its distinctive features, nanocellulose has been adopted by various industries for over a decade. It is a biodegradable, lightweight, strong nanofiber with high tensile strength, a large surface area, and versatile surface functionalization [6,7,8]. Additionally, nanocellulose presents a promising opportunity for the 3D printing technology. Among the various technique of 3D printing, liquid deposition modeling (LDM) is the most suitable, as the nanocellulose would be printed in liquid form [9,10]. This is due to its ability to maintain rigidity after being printed, which meets the essential criteria for LDM, such as solidification, shear-thinning properties, thixotropy, and gel formation [11,12,13].

3D printed materials for water remediation significantly enhance their capability and performance through unique physical appearances; whereas, the design of adsorbents plays a crucial role in adsorption and can be precisely controlled through 3D printing techniques. Design selection for adsorbents include a high surface area, with many pores to provide more active sites for the particle attachments on the surface. A monolithic design, with porous structures, enhances the performance of mass and heat transfer during adsorption [14,15]. Furthermore, the use of 3D printing techniques in the production of adsorbents is economically beneficial due to its faster production rate, high resolution, replication abilities, and the reusability of the equipment [14].

Therefore, in this work, cellulose nanofibrils (CNF) extracted from local lignocellulosic biomass was functionalized using a Fenton oxidation reaction of Fe(0) particles as a regeneration agent. The adsorption performance of the functionalized 3D printed nanocellulose was evaluated using a batch and fixed-bed column and compared to the adsorption behavior of neat nanocellulose. The adsorption mechanism of functionalized CNF was determined by analyzing data from both batch and fixed-bed adsorption studies using various adsorption models. In addition, the regeneration and recyclability of the 3D printed CNF columns were examined.

## 2. Materials and Methods

### 2.1. Materials

Cellulose isolation, as well as nanocellulose and Fe(0) preparation, were carried out using formic acid, FA (CH_2_O_2_), sodium hydroxide (NaOH), hydrogen peroxide (H_2_O_2_), iron(II) sulphate heptahydrate (FeSO_4_·7H_2_O), and sodium borohydride (NaBH_4_) from Merck, Darmstadt, Germany. The solutions of methylene blue trihydrate, MB (Merck, Darmstadt, Germany) were prepared and diluted to the necessary initial concentrations. Standard calibration curves were created using varied concentrations of MB solution, yielding absorbances ranging from 0.1 to 1. The pH of the solutions was adjusted to the required level with 0.1 M NaOH and 0.1 M HCl.

### 2.2. Nanocellulose Preparation

The cellulose extraction from oil palm empty fruit bunch (OPEFB) fibers was conducted according to the methods in a previous study [13]. In brief, 95% formic acid was utilized to separate lignin from OPEFB fibers at a weight ratio of 30:1. The remaining OPEFB pulp underwent purification through an alkaline–oxidation reaction using NaOH (2 wt.%) and H_2_O_2_ (2 wt.%) and was further treated with the catalytic oxidation of Fe(II). The purified cellulose was fibrillated with deionized water using a high-speed blender (Vitamix 5200, Vita-mix, Ohio, United States) at 37,000 rpm for 10 min. To prevent cellulose hydrolysis, the temperature during the fibrillation process was kept below 70 °C. 

To extend the life-cycle of CNF, the adsorbent was composited using Fe(0) particles as catalysts for the preparation of functionalized CNF. The preparation of the Fe(0) particles was conducted following the methods used in our previous work [16,17]. Ferrous iron from FeSO_4_·7H_2_O was mixed with the reducing agent NaBH_4_ in 100 mL of ethanol with a 1:2 weight ratio. The synthesized Fe(0) particles were blended with the defibrillated cellulose at 1 wt.% and kept at 4 °C until further use.

### 2.3. Parameter for Liquid Printing

The 3D printing procedure was carried out with a fabricated paste extruder and integrated with an Ultimaker 2+ 3D printer (Ultimaker, Utrecht, Netherlands). The extrusion was achieved using a tapered nozzle (15 G) with an internal diameter of 1.40 mm. Prior to printing, the printer’s build plate was cleaned with ethanol to make the produced sample easier to remove. To optimize the printing profile, the computer-aided design model was turned into an STL file that could be read by slicer software (Ultimaker Cura 4.3, Geldermalsen, The Netherlands). The samples were dried at room temperature until they reached a consistent weight, then stored in a desiccator for the adsorption study and detail characterization.

### 2.4. Adsorption Study

The adsorption kinetics study were conducted in a flask containing methylene blue, MB, and functionalized 3D printed CNF at the ratio of 1 g/L for 4 h under mechanical stirring at 200 rpm. The starting concentrations of pH and MB were changed from 3 to 9, and 50 to 300 mg/L, respectively. At the desired adsorption time, aliquots of the solution (0.1 mL) were acquired and centrifuged, and the concentration of MB was determine using a UV-spectrophotometer. The quantity of MB adsorbed at time *t*, *q*_*t*_ (mg/g), was calculated using the equation from our previous work:

(1) $$q_{t}=\frac{\left(C_{0}-C_{t}\right)V}{m},$$

where *C*_0_ represents the initial concentration and *C*_*t*_ represents the MB concentration (mg/L) at time *t* (min) [18]. The volume of the MB solution is given in liters, and the mass of the adsorbent is given in grams.

The adsorption isotherm study was carried out in a water bath shaker at a constant speed of 150 rpm, with functionalized 3D printed CNF added to 100 mL of MB at various concentrations (100, 150, 200, 250, 300, and 350 mg/L) and varied temperatures (20, 40, and 60 °C). Using the following equation, the quantities adsorbed at equilibrium, *q*_*e*_, were estimated from the equilibrium concentration of MB, *C*_*e*_ [18].

(2) $$q_{e}=\frac{\left(C_{0}-C_{e}\right)V}{m},$$

The functionalized 3D printed CNF column adsorption study were conducted in a designated fixed-bed column packed with the adsorbents. At a constant pH, continuous adsorption experiments were carried out with varied doses of MB (200, 300, and 400 mg/L) in the influent solution. A flowmeter was used to regulate and vary the flow rate of the influent to obtain different flow rates (5, 10, and 15 mL/min). To assess the time necessary for breakthrough, the concentration of MB in effluents was monitored.

After the breakthrough of adsorption with 300 mg/L of MB in the packed functionalized 3D printed CNF, column desorption tests were performed. A CH_3_COOH desorbing solvent was fed into the packed column until the MB content in the effluent was almost zero. To improve the regeneration of packed adsorbent, the functionalized CNF was immersed in 40 mM of H_2_O_2_ for 24 h and rinsed with deionized water for the removal of excess MB. To assess the performance of the packed 3D printed CNF, regeneration tests were carried out for up to five cycles for adsorption regeneration. The quantity of dye desorbed was determined by spectrophotometry using Equation [18].

(3) $$\mathrm{Amount}\;\mathrm{of}\;\mathrm{dyes}\;\mathrm{desorbed}\left(\%\right)=\frac{\mathrm{Concentrationdesorbed}\left(\mathrm{mg}/L\right)}{\mathrm{Concentrationadsorbed}\left(\mathrm{mg}/L\right)}\times 100\%,$$

### 2.5. Characterization

The morphological structure of the printed samples was examined using a field emission scanning electron microscope (FESEM) (Merlin Compact, Zeiss Pvt Ltd., Oberkochen, Germany). MB trihydrate solutions (Stance, London, UK) were produced and diluted to the desire concentrations. A UV–Vis spectrophotometer (Single beam UV spectrophotometer SP-UV 300SRB, Spectrum Instruments GmbH, Überlingen, Germany), with a max of 665 nm, was used to quantify the concentrations of MB in the working solutions.

## 3. Results

### 3.1. Characterization

**Figure 1:**
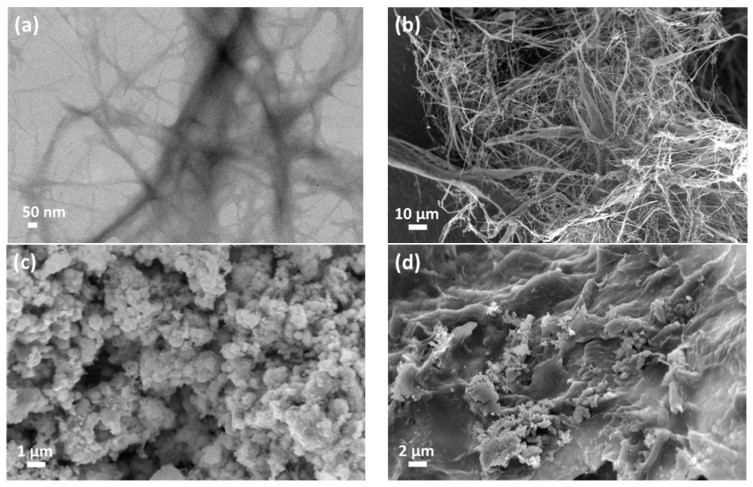
Morphology of (**a**) CNF on TEM and (**b**) CNF, (**c**) Fe(0), and (**d**) functionalized CNF with Fe(0) under the micrograph of FESEM.

The morphological structure of both CNF and Fe(0) particles were investigated under TEM and FESEM micrographs, as shown in Figure 1. Figure 1a shows that the fibrillated cellulose has been obtained with the desire diameter of ~15 nm, comparable with that obtained in the previous work [18]. A similar orientation of CNF can be observed under the FESEM micrograph (see Figure 1b); whereas, the aggregation of Fe(0) particles was exhibited in a chain-like structure, with the diameter in the range of 45–170 nm (see Figure 1c). The wide aggregation of the Fe(0) particles was expected as a result of oxidation reactions with iron oxide and hydroxide oxide [16]. However, since the cellulose was easily agglomerated due to the dehydration, the formation of flakes can be observed in Figure 1d. 

**Figure 2:**
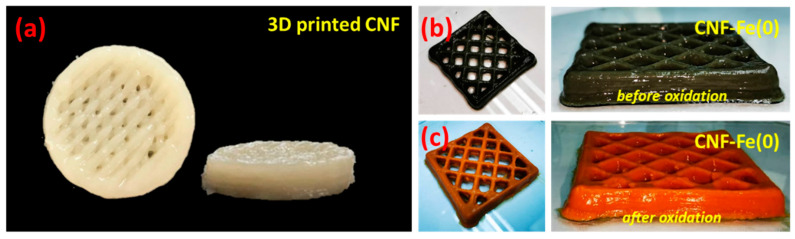
3D printed structures of (**a**) CNF, (**b**) functionalized CNF with Fe(0) before oxidation, and (**c**) after oxidation reaction.

Although the formulation of nanocellulose was modified with Fe(0) particles, the parameter used for liquid printing managed to construct the 3D printed structure that comparable with our previous work (see Figure 2) [13]. The catalyst added in the system clearly indicates that the storage modulus (G′) remains greater than loss modulus (G″). The rheological factor retains the significant factor of maintaining the shape of 3D printed CNF, as demonstrated in Figure 2b,c. It is seen that even though the Fe(0) particles may weaken the polymers, the structure remains intact after the oxidation reaction.

### 3.2. Batch Adsorption Study

**Figure 3:**
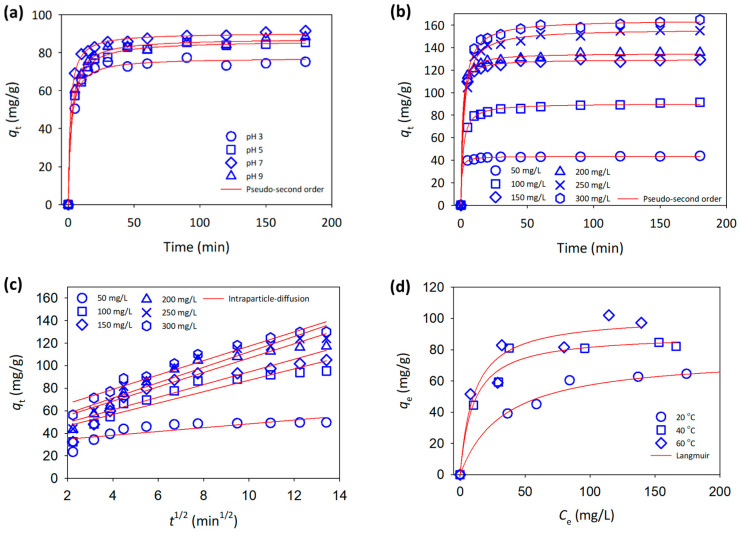
Adsorption models of MB onto 3D printed CNF for (**a**) different initial pH, (**b**) pseudo-second order, (**c**) intraparticle diffusion, and (**d**) Langmuir isotherm.

At an initial MB concentration of 100 mg/L, the effect of the initial pH resulted in the adsorption capacity of the 3D printed CNF increasing at pH 3 to 7 and subsequently, reducing at pH 9 (see Figure 3a). The presence of hydroxyl and carbonyl groups in the cellulosic materials would have strengthened the electrostatic interaction with the positively charged ion. The degree of ionization (protonation) of the adsorbent is affected by the pH of the solution [19]. Because of the low surface charges of the adsorbent, the adsorption capacity was modest at pH 3. Furthermore, at low pH, H+ ions will compete with MB molecules for adsorption [20]. The presence of negative charges on the adsorbent surface increased the electrostatic attraction between the dyes and the adsorbent as the pH of the solution increased, resulting in a maximum adsorption capacity at pH 7 [21]. Due to the demethylation of MB in alkaline solution, increasing the pH to 9 lowered the adsorption capacity marginally [22].

The pseudo-first order and pseudo-second order adsorption kinetics models were utilized to match the results of the experiments [23,24]. The formulae of these kinetic models are:

(4) $$\ln\;(q_{e}-q_{t})=\ln\;q_{e}-k_{1t}$$

(5) $$\frac{t}{q_{t}}=\frac{1}{k_{2}q_{e}^{2}}+\frac{1}{q_{e}}t$$

where *q*_*e*_ and *q*_*t*_ are the quantities of MB adsorbed per mass of adsorbent at equilibrium and over time, respectively, and *t* is the temperature (min). The pseudo-first order and pseudo-second order models have rate constants of *k*_1_ and *k*_2_, respectively. The pseudo-first and pseudo-second order non-linearized plots are shown in Figure 3a,b. The correlation coefficient of the pseudo-second order model is greater than that of the pseudo-first order model (see Table 1).

The following equation [25] was used to estimate the diffusion process using the intraparticle diffusion model:

(6) $$q_{t}=k_{i}t^{\frac{1}{2}},$$

where *k*_*i*_ is the intraparticle diffusion constant, which can be calculated by plotting *q*_*t*_ vs. *t*_1/2_ (Figure 3c). Instantaneous surface adsorption, intraparticle diffusion, and ultimate equilibrium are the three main processes in intraparticle diffusion. From the plot, two conclusions may be drawn. Intraparticle diffusion is the rate-controlling step, if the straight line passes through the origin. Other processes, in addition to intraparticle diffusion, may be involved in the adsorption process. The adsorption process progressed linearly throughout the entire duration, and the intercept was non-zero at lower MB concentrations, but at higher MB concentrations, multiple lines were observed, suggesting that mass transfer effects from boundary layer diffusion and intraparticle diffusion effects in the adsorbent’s micro and mesopores were present [26].

To match the adsorption experimental data, both Langmuir and Freundlich adsorption isotherms were adopted. Langmuir isotherms imply monolayer coverage of the adsorbate over a homogenous adsorbent surface, with equal adsorption activation energy for each molecule adsorbed on the surface, and Freundlich isotherms describe reversible adsorption and are not limited to monolayer formation [27,28]. The Langmuir (7) and Freundlich (8) isotherms can be written as follows:

(7) $$q_{e}=\frac{Q_{0}bC_{e}}{1+bC_{e}}$$

(8) $$q_{e}=K_{F}C_{e}^{1/n}$$

where *q*_*e*_ represents the amount of MB adsorbed per mass of the adsorbent at equilibrium (mg/g), *C*_*e*_ represents the equilibrium concentration of MB in the solution (mg/L), *Q*_0_ represents the maximum adsorption capacity per unit weight of the adsorbent (mg/g), and *b* represents a constant related to the energy of the adsorption (L/mg) [27,28]. The Freundlich constants are *K*_*F*_ and 1/n. The relative adsorption capacity of the adsorbent is represented by *K*_*F*_, and the degree to which adsorption is dependent on the equilibrium MB concentration is represented by *n* [18].

(9) $$R_{L}=\frac{1}{1+bC_{0}}$$

With the experimental results shown in Figure 3d, the non-linearized Langmuir model closely matched with the experimental results for MB adsorption using functionalized 3D printed CNF, with an excellent correlation coefficient, *r*^2^ 0.99, which was better than that for the Freundlich isotherm (see Table 2). This might be owing to the uniform distribution of adsorption sites on the CNF surface, even with the addition of Fe(0) particles. The Langmuir isotherm revealed that the *Q*_0_ increased with increasing temperatures, with the greatest *Q*_0_ of 101.7 mg/g for the adsorption at 60 °C. The adsorption of MB was slightly lower than that in a previous work, which managed to uptake MB up to 122 mg/g using neat CNF. It was expected that with the addition of Fe(0) particles into the system, the allocation of the surface active sites would slightly reduce the adsorption performance [16].

To evaluate the effect of temperature on MB uptake, the thermodynamic parameters were calculated using the Van’t Hoff equations;

(10) $$\ln K_{d}=\frac{\Delta S{}^{\circ}}{R}-\frac{\Delta H{}^{\circ}}{RT}$$

(11) $$\Delta G{}^{\circ}=-RT\;\ln\;K_{d}$$

where *R* is the ideal gas constant, *T* is the temperature (K), and *K*_*d*_ is the distribution coefficient [29]. The thermodynamic parameters were determined by calculating the slope and intercept of the adsorption plot at 300 mg/L at various temperatures. Positive values of ∆*H*° (11.49 kJ/mol) and ∆*S*° (23.78 J/mol K) indicate an endothermic process, increasing disorder at the solid–solution interface during adsorption. The negative value of ∆*H*° (from −45.83 to −36.50 kJ/mol) at higher temperatures suggests a spontaneous adsorption reaction [30].

### 3.3. Column Adsorption Study

**Figure 4:**
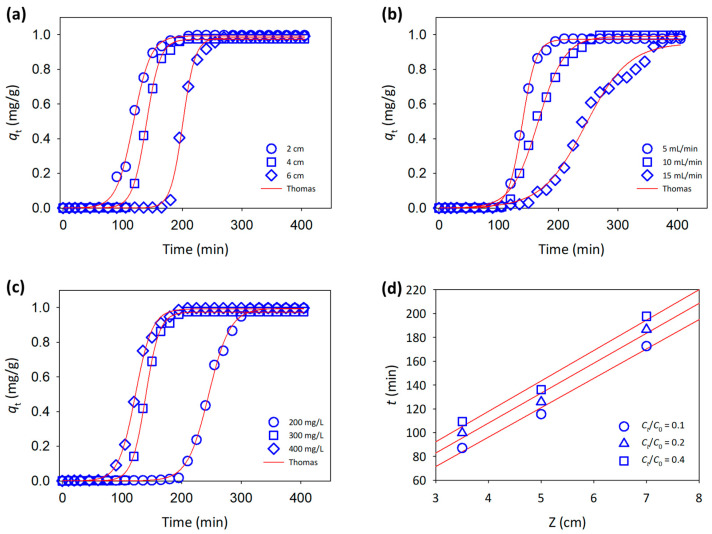
Adsorption of MB onto a functionalized 3D printed column with different controlled parameters: (**a**) different column bed heights, (**b**) different influent flow rate, (**c**) different initial influent concentration, and (**d**) linearized BDST model fit at different service times.

With a controlled flow rate (15 mL/min) and MB concentration (300 mg/L), the adsorption performance of fixed-bed 3D printed columns was investigated, and the temperature and pH were maintained at 20 °C and 7, respectively. The greatest bed height resulted in the longest time for MB adsorption to reach breakthrough, as seen by the breakthrough curves in Figure 4a. The contact duration between MB and the adsorbent was extended when the bed height of the column was raised, lowering the adsorption response rate and resulting in higher MB adsorption [29].

The BDST model was used to match the experimental data collected at various bed heights, which is based on physically measuring the bed’s capacity at various breakthrough percentage values (Figure 4d). Fitting this model to the data can assist in forecasting the bed’s adsorbent performance in response to changes in the column adsorbent [29]. The BDST model is written as follows:

(12) $$t=\frac{N_{0}Z}{C_{0}v}-\frac{1}{k_{B}C_{0}}\ln\left(\frac{C_{0}}{C_{t}}-1\right)$$

where *k*_*B*_ is the adsorption rate constant (L/mg·min), *N*_0_ is the adsorption capacity (mg/L), *Z* is the bed height (cm), and *v* is the linear velocity (cm/min). The experimental data were plotted as a function of bed height, *Z* versus time *t*, and the service time for 10, 20, and 40% values of *C*_*t*_/*C*_0_, yielding *k*_*B*_ and *N*_0_, as summarized in Table 3. These results show that increasing the value of *C*_*t*_/*C*_0_ will increase the adsorption capacity while decreasing the rate constant of the adsorbent.

The flow rate of the MB solution through the column significantly affected the adsorption and breakthrough curves. Figure 4b shows the results from different flow rates with a constant MB concentration, pH, and temperature. Lower flow rates increase the time required for the adsorption of MB to reach the breakthrough point. This increase is due to the longer contact time of the adsorbate with the column surface sites, improving the distribution of the adsorbate influent [31]. At lower flow rates of the MB solution, the adsorbate will homogeneously occupy the surface sites of the adsorbent and slowly saturate all sites down to the packed column. Increasing the flow rate of the influent leads to some heterogeneously saturated packed adsorbent, resulting in a lower adsorption capacity and a shorter time required to reach the breakthrough point.

Figure 4c shows the effect of the MB concentration on the time taken to reach breakthrough. The initial MB concentration provides an important driving force to overcome all mass transfer resistance between the aqueous phase and the solid phase. The lower MB concentration of 200 mg/L can stand for almost 4 h, in contrast with the higher MB concentrations. This can be explained by the interaction with MB through the boundary layer effect, followed by its diffusion to the boundary layer film and finally, to the porous structure of the adsorbent [18]. The lower MB concentration facilitated the interaction with unoccupied surface sites, resulting in higher adsorption yields. The operation time is much shorter at higher MB concentrations because of the saturation of the surface sites [32].

The experimental column adsorption results were fit to the Thomas expression, as this model assumes the Langmuir model adsorption-desorption, no axial dispersion, and a rate driving force that obeys pseudo-second order reversible reaction kinetics [33]. The linearized equation of the model can be expressed as follows:

(13) $$\ln\left(\frac{C_{0}}{C_{t}}-1\right)=\frac{k_{\mathrm{Th}}q_{0}m}{F}-\frac{k_{\mathrm{Th}}C_{0}V_{\mathrm{eff}}}{F},$$

where *k*_*Th*_ is the Thomas rate constant (L/mg·min), *q*_0_ is the maximum solid phase concentration (mg/g), m is the mass of the adsorbent in the column (g), *V*_*eff*_ is the volume of the effluent (L), and *F* is the flow rate of the influent (L/min). The values of *k*_*Th*_ and *q*_0_ were calculated and are summarized in Table 4. The experimental data fit the Thomas model well, as the *r*^2^ value is ~0.90 for all parameters, complementing the Langmuir isotherms for the batch isotherm studies. The predicted *q*_0_ values in the batch and column studies were close. However, the value of *q*_0_ in the column is 128.04 mg/g, which was slightly higher than that in the batch adsorption studies and comparable to that in the previous study [6,18]. This suggests that the MB adsorbed through packed CNF was saturated onto the porous sites of CNF, allowing the following MB molecules to accumulate between the adsorbent surfaces. 

**Figure 5:**
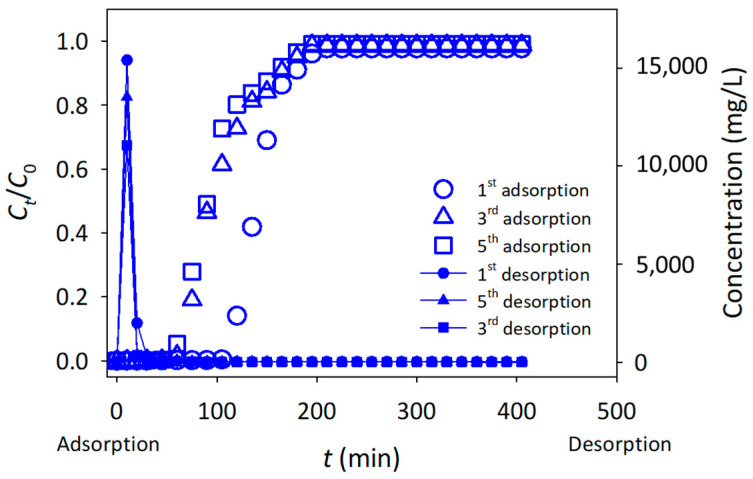
Fixed-bed column regeneration for five cycles of adsorption-desorption.

Desorption studies indicate that the maximum amount of dye desorbed from the CNF is 83.0%, using the CH_3_COOH solution. These suggest that acid, such as CH_3_COOH, is required to release MB from CNF, based on ion exchange and electrostatic attraction [34]. Regeneration studies were conducted by performing up to five cycles of fixed-bed column adsorption-desorption, using CH_3_COOH solution as the desorbing solvent. The performances of the packed adsorbent in the column were analyzed for each regeneration cycle by monitoring the service time (breakthrough). Figure 5 shows that, although the adsorption of MB after 5 cycles can still achieve a 99% removal rate, after the first adsorption cycle, the service time of the column is reduced significantly. Besides, the adsorption performance of the column decreased from 102.08 mg/g to 81.38 mg/g for the first and second adsorption cycles, respectively. Furthermore, the service time and adsorption performance of the column were maintained after the third and subsequent cycles. These results suggest that the nanosizes of CNF, which were difficult to be desorbed by CH_3_COOH, were occupied by the MB molecules during the first adsorption cycle; however, after regeneration by the Fenton oxidation process, the numbers of surface-active sites was recovered, providing for the uptake of MB for next cycle of adsorption.

## 4. Conclusions

In this study, cellulose from OPEFB fibers was successfully transformed into nanocellulose and functionalized with Fe(0) particles for water remediation. Adsorption techniques, including batch and fixed-bed column methods, were carried out to gain insight into the adsorption mechanism. The results were then used to create a functionalized 3D-printed nanocellulose fiber (CNF) adsorbent. The Langmuir and Thomas isotherm models showed that the adsorption of MB onto the adsorbent was monolayered and homogenous, with a maximum estimated capacity of 128.04 mg/g, slightly higher than that of batch adsorption. The functionalized 3D-printed CNF adsorption showed significant improvement, particularly in the regeneration of the column adsorbent, due to the degradation of MB by Fe(0) on the surface of CNF. The results of this study are promising for the development of an adsorbent using additive manufacturing because of its versatility and selective design.

## Figures and Tables

**Table 1:** Adsorption kinetic model data from different initial concentrations.

Initial MB Concentration (mg/L)		Pseudo-First Order			Pseudo-Second Order			Intraparticle Diffusion		
	*q*_*e* exp_ (mg/g)	*q*_*e* cal_ (mg/g)	*k*_1_ (h^−1^)	*r* ^2^	*q*_*e* cal_ (mg/g)	*k*_2_ (g/mg·min)	*r* ^2^	*q*_*e* cal_ (mg/g)	*k*_*i*_ (mg/g·h^1/2^)	*r* ^2^
50	44.11	42.76	0.254	0.962	43.45	0.0221	0.994	41.05	0.228	0.733
100	89.87	86.33	0.149	0.879	89.88	0.0135	0.976	60.60	2.203	0.747
150	108.70	132.43	0.285	0.935	134.01	0.0099	0.951	68.68	2.667	0.908
200	117.80	130.28	0.223	0.800	133.93	0.00043	0.892	72.59	2.935	0.967
250	130.33	149.41	0.119	0.927	156.79	0.00015	0.956	61.43	4.891	0.948
300	155.43	158.61	0.100	0.944	167.58	0.00011	0.990	86.86	4.989	0.726

**Table 2:** Calculated equilibrium constants for the adsorption of MB onto functionalized CNF.

Temperature	Langmuir Model				Freundlich Model		
(°C)	*Q*_0_	*b*	*R_L_*	*r^2^*	*K*_*F*_	*n*	*r* ^2^
20	76.90	0.211	0.013	0.996	35.5	4.65	0.973
40	89.87	0.228	0.034	0.995	36.7	4.72	0.930
60	101.70	0.233	0.019	0.993	36.9	3.89	0.923

**Table 3:** Calculated data experiment conducted in the BDST model.

*C*_t_/*C*_0_	*k*_*B*_ (L/mg·min)	*N*_0_ (mg/L)	*r* ^2^
0.1	259.93	374.94	0.988
0.2	90.63	384.87	0.980
0.4	42.81	388.89	0.980

**Table 4:** Adsorption data from the Thomas model of the column adsorption of MB onto functionalized CNF.

*F* (L/min)	z (cm)	*C*_0_ (mg/L)	*k*_*Th*_ (L/mg·min)	*q*_0 *Th*_ (mg/g)	*r* ^2^
0.005	4	300	0.86 × 10^−4^	128.04	0.965
0.010	4	300	2.14 × 10^−4^	102.08	0.950
0.015	4	300	5.29 × 10^−4^	62.50	0.950
0.010	2	300	4.37 × 10^−4^	66.19	0.964
0.010	4	300	2.14 × 10^−4^	102.08	0.950
0.010	6	300	1.43 × 10^−4^	113.88	0.908
0.010	4	200	1.89 × 10^−4^	108.39	0.901
0.010	4	300	2.14 × 10^−4^	102.08	0.950
0.010	4	400	1.51 × 10^−4^	119.33	0.973

## Data Availability

Not applicable.
